# Valorizing sardine scales: a circular approach to sustainable collagen for cosmetics and nutrition applications

**DOI:** 10.3389/fphar.2024.1443358

**Published:** 2024-11-06

**Authors:** Marcia Santos Filipe, Rebeca André, Marco Ferreira, Ana María Diaz-Lanza, Vânia André, Marta M. Alves, Rita Pacheco, Patrícia Rijo

**Affiliations:** ^1^ Escola de Ciências e Tecnologias da Saúde (ECTS), CBIOS- Universidade Lusófona’s Research Center for Biosciences and Health Technologies, Lisbon, Portugal; ^2^ Departamento de Ciencias Biomédicas (Área de Farmacología; Nuevos Agentes Antitumorales, Acción Tóxica sobre Células Leucémicas), Facultad de Farmacia, Universidad de Alcalá de Henares, Alcalá de Henares, Madrid, Spain; ^3^ Pinhais and Cia, Lda, Matosinhos, Portugal; ^4^ Centro de Química Estrutural, Institute of Molecular Sciences, Universidade de Lisboa, Lisboa, Portugal; ^5^ Departamento de Engenharia Química, Centro de Química Estrutural, Institute of Molecular Sciences, Instituto Superior Técnico, Universidade de Lisboa, Lisboa, Portugal; ^6^ Department of Chemical Engineering, ISEL - Instituto Superior de Engenharia de Lisboa, Lisboa, Portugal; ^7^ Faculdade de Farmácia, Instituto de Investigação do Medicamento (iMed.ULisboa), Universidade de Lisboa, Lisbon, Portugal

**Keywords:** sardine scales, collagen, circular economy, natural compounds, skin products

## Abstract

**Background and Objective:**

In recent years, the consumption of fish products has led to a worrying trend where approximately two-thirds of the total amount of fish is discarded as waste. At the same time, scientific interest in exploring natural collagen sources for cosmetics and dietary supplements has increased. This study explores the potential of valorizing sardine scales (*Sardina pilchardus*), a by-product of the canning industry, through the extraction of collagen for potential use in dermocosmetic formulations and food supplements.

**Methods:**

Collagen from sardine scales was obtained though acid and enzymatic extraction. The collagen extracts were characterized by UV-Vis, FTIR spectroscopy, SDS-PAGE, powder X-ray diffraction (PXRD) and scanning electron microscopy (SEM). The collagen was hydrolysed with papain to small peptides. Subsequently, the biological activities of acid-soluble collagen as well as the collagen peptides in terms of antioxidant and antimicrobial activity were evaluated. Furthermore, the capacity of collagen peptides to permeate the intestinal barrier, simulated with caco-2 cells, was evaluated.

**Results:**

Purified collagen extracts were obtained from sardine scales, with enzymatic extraction method having a yield three times higher than the acid method. The SDS-PAGE analysis confirmed the extraction of type I collagen as well as its hydrolysis into small fragments (25–12 kDa). In terms of biological activities, collagen and collagen peptides have not demonstrated antimicrobial activity. However, regarding antioxidant activity, collagen peptides showed three times more capacity compared to non-hydrolyzed collagen. Meanwhile, in 6 h, about 6.37% of collagen peptides could permeate the intestinal barrier.

**Conclusion:**

This work represents a continuous effort to advance our understanding and utilization of Portuguese marine waste resources, with focus on the valorization of sardine co-products for the development of food supplement or cosmetic formulations, contributing to the sustainable evolution of the circular blue economy.

## 1 Introduction

Over 70% of the Earth’s surface is covered by seas and oceans, which represents and plays a pivotal role in global economies, particularly the fisheries industry. However, intensive exploitation of marine resources has led to significant production of waste. According to the Food and Agriculture Organization (FAO), in 2018, approximately 12% of the total fish production, reaching 179 million tons, was directed to non-food purposes, raising concerns about the environmental and economic impact of such waste (Rudovica et al., 2021). Despite the recognized benefits of fish products for a balanced diet and healthy lifestyle, nearly two-thirds of the total fish harvested ends up discarded as waste, with scales, viscera, heads, and tails constituting the major by-products (Coppola et al., 2021).

Marine biomass, comprising a rich and diverse array of natural products, holds immense potential due to its varied composition and functional properties, which include bioactive compounds, such as polyphenols, peptides, and polysaccharides. These compounds exhibit both biocompatibility and diverse bioactivities, making them ideal candidates for the development of renewable, low-cost, and abundant bioactive materials (Manivasagan et al., 2017). Sardine consumption, particularly in Europe, has been steadily increasing, with sardine waste becoming a focal point for the canning industry in terms of valorising unused biomass (The European market potential for, 2021).

Research has highlighted the potential of sardine by-products, such as *Sardina pilchardus* scales, as promising sources of chitin and chitosan (Aboudamia et al., 2020). Additionally, a separate study on the scales of *Sardinella longiceps* have revealed a high concentration of collagen (Muthumari et al., 2016). Studies on other fish species, like Tilapia (*Oreochromis niloticus*), have also shown that the collagen in its skin possesses unique physicochemical properties, making it valuable for biomedical and dermocosmetics applications (Reátegui-Pinedo et al., 2022; Sánchez-Tuesta et al., 2021).

The growing demand for sardines has prompted exploration into various methods of valorising sardine by-products, not only for sustainable energy and feed (Bahammou et al., 2019), but also for extracting high-value bioactive compounds such as collagen, chitin, enzymes, gelatine, glycosaminoglycans, polyunsaturated fatty acids (PUFA), minerals, vitamins, and antimicrobial peptides.

Collagen, a major structural protein in connective tissues (Venkatesan et al., 2017), is particularly noteworthy for its applications in food supplements and cosmetics, once has been described with capacity to prevent signs of aging and improving skin conditions such as brightness, firmness, hydration, among other factors (Chen et al., 2018; León-López et al., 2019a). Collagen hydrolysates are also used in seasonings, non-allergic preservatives for drugs, ingredients for dietary materials, and parenteral nutrition products (Nakayama et al., 2000), as well as antioxidant and antimicrobial agents (Gómez-Guillén et al., 2011; Lima et al., 2015).

Recent studies have highlighted the remarkable benefits of using fish skin in treating burns and amputation patients, revealing the untapped potential of fish by-products (Magnusson et al., 2017; Lau et al., 2019). Biologic materials derived from the extracellular matrix (ECM) of human or animal tissues are commonly used as scaffolds to support tissue and organ regeneration. Decellularization, a process that removes cells from a tissue or organ, leaves behind an acellular ECM rich in structural and functional proteins that promote cell proliferation and tissue regeneration. There is increasing interest in using decellularized tissues from non-mammalian sources due to concerns over religious restrictions and the risk of zoonotic diseases. Marine-derived ECMs, in particular, offer a promising alternative because of their structural and functional similarities to mammalian ECMs. Additionally, the seafood industry generates a substantial amount of waste—about 75% of fish weight—including skins, scales, bones, and internal organs. Repurposing these underutilized resources into valuable products could enhance revenue and reduce waste (Lau et al., 2019).

In Europe, these findings contribute to a broader understanding of marine-derived compounds’ potential across various industries. Recognizing the importance of addressing waste in the marine industry, the concept of a circular economy has gained momentum. The Portuguese company “Conservas Pinhais & Cia, Lda.”, primarily focused on sardines, discards significant biomass as waste, including fish scales. In an attempt to redirect this waste into novel uses, the extraction of bioactive compounds from these by-products has become a priority, leading to the discovery of a plethora of compounds with potential applications in human consumption, pharmaceuticals, and dermocosmetics (Rudovica et al., 2021; Khawli et al., 2019).

In this context, this study explores the collagen extraction derived from the scales of *S. pilchardus* with the addition of an acid solution and the aid of pepsin enzyme. The biological activities and characterization of the extracted collagen were thoroughly evaluated. The findings of this research seek to enhance the value of sardine scales, transforming this fishery by-product into a valuable resource while promoting environmental sustainability. Additionally, the study provides foundational data that could inform and guide future research in this area.

## 2 Materials and methods

### 2.1 Reagents

Methanol and Acetonitrile of HPLC-grade were purchased from VWR Chemicals (Fontenay-sous-Bois, France). Trifluoroacetic acid (TFA), Glacial acetic acid, tris(hydroxymethyl)aminomethane (Tris) and sodium chloride were purchase from Merck (Darmstadt, Germany). SnakeSkin™ Dialysis Tubing 10 k MWCO, 22 mm, Thermo Scientific. Ethanol 96% was bought from Carlo Erba (Peypin, France).

2,2-diphenyl-1-picrylhydrazyl (DPPH), quercetin, papain from papaya latex (≥ 10 U/mg protein), caffeine, pepsin from porcine gastric mucosa (≥ 250 units/mg solid), 3-(4,5-dimethylthiazol-1-yl)-2,5-diphenyltetrazolium bromide (MTT), sodium dodecyl sulfate (SDS), Bovine Serum Albumin (BSA) and Mueller-Hinton broth were obtained from Sigma (Barcelona, Spain). Sabouraud agar was purchased to Biokar Diagnostics (Allone, France).

Roswell Park Memorial Institute (RPMI) medium, fetal bovine serum (FBS), pen-strep (penicillin 100 U/mL and streptomycin 100 U/mL), trypsin (10 ×) and L-glutamine (2 Mm), Phosphate-Buffered Saline (PBS) and Hanks balanced salt solution (HBSS) were purchase to Lonza (Basel, Switzerland). Coomassie Brilliant Blue R-250 was supplied by Bio-Rad (Hercules, CA, United States). Mini Protein Gel Nu-PAGE™ 4 to 12% Bis-Tris, Bolt^®^ MOPS SDS Running Buffer (20 ×) were obtained from Thermo Fisher Scientific (Waltham, MA, United States). Protein marker NZYBlue and 5 × SDS-PAGE Sample Loading Buffer were purchased from NZYTech (Lisbon, Portugal).

### 2.2 Fish scales

The fish scales from *S. pilchardus* were obtained from the Portuguese company “Conservas Pinhais & Cia, Lda.” located in Matosinhos. The scales were frozen and, prior to extraction, underwent a thorough cleansing with tap water to ensure the removal of any residual blood and skin.

### 2.3 Collagen extraction

#### 2.3.1 Acid soluble collagen (ASC) and pepsin soluble collagen (PSC)

Firstly, to remove non collagen proteins the scales were washed with 10% NaCl (w/v) and then the process of demineralization was carried out following the protocol outlined in Muthumari et al. (2016). Sardine scales were extracted with 0.5 M acetic acid, with a ratio of 1:10 (w/v) over a 4-days period. Then the scales were filtered and transferred to a separated conical flask, followed by centrifugation at 8,000 × *g* for 30 min. After centrifugation, the supernatant was salted out by adding NaCl to reach a final concentration of 2.5 M in the presence of 0.05 M tris(hydroxymethyl) aminomethane (pH 7.0) (Ahmad et al., 2010) and was left undisturbed overnight. During this phase, the collagen protein was isolated by precipitation. The precipitated collagens were separated by centrifugation at 8,000 × *g* for 30 min (Muthumari et al., 2016). The resulting precipitated collagen were dissolved in 0.5 M acetic acid and subsequently dialyzed against 10 volumes of 0.1 M acetic acid and distilled water for 24 h, respectively. The collagen extract was lyophilized, identified as “Acid soluble collagen” (ASC) extract and stored at 4°C until analysis.

To obtain the “Pepsin soluble collagen” (PSC) extract the scales residues from the acid extraction were suspended in 0.5 M acetic acid with 0.5% (w/v) pepsin for 72 h. The following extraction steps of PSC extract were the same as the extraction of ASC (Zhang et al., 2011). All procedures were performed at 4°C.

### 2.4 Characterization of collagen extracts

#### 2.4.1 Ultraviolet (UV)-Vis spectroscopy

The UV-Vis analyses of ASC and PSC extracts (1 mg/mL) were conducted following the procedure outlined by Srinivasan and Durairaj (2021).

#### 2.4.2 Fourier transforms infrared spectroscopy (ATR-FTIR)

The FTIR spectra of ASC and PSC extracts were obtain using the PerkinElmer^®^ Spectrum TWO (PerkinElmer Inc, Waltham, MA, United States) equipped with an attenuated total reflectance (ATR) device. The procedure follows the method described by Srinivasan and Durairaj (2021).

#### 2.4.3 Powder X-ray diffraction (PXRD)

The powder X-ray patterns for ASC and PSC extracts were performed using CuKα radiation source on a D8 Advance Bruker AXS θ-2θ diffractometer (40 kV, 40 mA) equipped with a LYNXEYE-XE detector and a Ni filter. Data was collected in the 4 ≤ 2θ ≤ 60° range with a step size of 0.02°. Crystallography Open Database (COD) was used to identify the presence of NaCl in the samples.

#### 2.4.4 Scanning electron microscopy (SEM)

For the morphological analysis of ASC and PSC extracts, scanning electron microscopy (SEM) analysis was performed with Phenom ProX G6 (Thermo Fischer Scientific).

### 2.5 Enzymatic hydrolysis of acid soluble collagen extract

The collagen hydrolysis was performed from ASC extract according to protocol by Hema et al. (2017) with some modifications. Briefly, ASC extract was dissolved in distilled water at 10 mg/mL and incubated at 60°C for 5 min. Then ASC extract was incubated with papain dissolved in distilled water in a ratio of 4.5% E/S, pH 6.3 at 55°C during 4.25 h, according to the optimal reaction temperature conditions described by Chen et al. (2023). The hydrolysis products (named collagen peptides) were subjected to a 95°C water bath for 10 min and then it was centrifuged at 10,000 × *g* for 10 min. The supernatant was freeze-dried and kept at 4°C until further analysis.

### 2.6 Sodium dodecyl sulfate–polyacrylamide gel electrophoresis (SDS-PAGE)

The samples of ASC extract and collagen peptides were dissolved in acetic acid 0.5 M, and the total protein in each sample was quantified using the modified Bradford method for collagen rich samples, described in López et al. (1993).

The SDS-PAGE analysis was performed as described in André et al. (2023). The samples of ASC extract and collagen peptides, containing 20 ug of protein, were loaded onto NuPAGE 4 to 12% gradient gels (Invitrogen™, Waltham, MA, United States) using a Mini Gel Tank (Invitrogen™). The conditions of electrophoresis followed the manufacturer’s instructions. The gels were stained for 1 h with a solution of 40% Coomassie R-250 blue, 50% methanol and 10% glacial acetic acid and subsequently distaining overnight in a solution of 7.5% glacial acetic acid, 10% ethanol and 82.5% distilled water.

Finally, the gels were photographed using ImageQuant LAS 50 (GE Healthcare Life Sciences^®^, Chicago, IL, United States), and analyzed using ImageJ software (NIH, Bethesda, MD, United States).

### 2.7 Biological activities of collagen

#### 2.7.1 Antioxidant capacity

The antioxidant potential of ASC extract and collagen peptides was evaluated through the 2,2-diphenyl-1-picrylhydrazyl (DPPH) radical method as described by Hernández-Ruiz et al. (2023). ASC extract and collagen peptides were tested at final concentration of 5 mg/mL, while the positive control, quercetin, was tested at 0.1 mg/mL. The results were expressed in percentage (%) of antioxidant activity.

#### 2.7.2 Antimicrobial activity

The antimicrobial activity of the ACS extract and collagen peptides was assessed using the well diffusion method, following the guidelines of the Clinical and Laboratory Standards Institute (CLSI) (CLSI, 2023). The samples were tested against Gram-negative bacteria (*Pseudomonas aeruginosa* ATCC 27853 and *Escherichia coli* ATCC 25922), Gram-positive bacteria (*Staphylococcus aureus* ATCC 25923 and ATCC 6538, *Staphylococcus epidermidis* ATCC 12228) and yeast strains (*Saccharomyces cerevisiae* ATCC 2601 and *Candida albicans* ATCC 10231). Samples were reconstituted in distilled water to a concentration of 10 mg/mL concentration. Positive controls consisted of reference antibiotics at 1 mg/mL: vancomycin for Gram-positive bacteria, norfloxacin for Gram-negative bacteria and nystatin for yeasts strains. Distilled water was used as the negative control.

Agar plates (Mueller–Hinton agar for the bacteria and Sabouraud agar for the yeasts) were inoculated with a standardized inoculum solution of each microorganism, corresponding to a 0.5 McFarland standard solution. Small wells (approximately 5 mm in diameter) were then made in the solid medium, where 50 μL of the samples and controls were placed. The Petri dishes are incubated at 37°C for 24 h. The method was tested in triplicate.

### 2.8 Cellular assays

#### 2.8.1 Cell culture

Caco-2 cells (ATCC#HTB37), a human colorectal adenocarcinoma epithelial cell line, were cultured in RPMI supplemented with 10% FBS, 100 U/mL penicillin, 100 U/mL streptomycin, and 2 mM L-glutamine in T25 flasks at 37°C in an atmosphere with 5% CO_2_. The culture medium was changed every 48–72 h.

#### 2.8.2 Determination of cytotoxicity

The cytotoxicity effect of collagen peptides (0.1–1 mg/mL) in Caco-2 cells was evaluated by MTT viability test as previously described (André et al., 2019). The cytotoxicity assays were done in 3 × 2 replicates for each concentration of sample. 3% of SDS was used as a positive control.

#### 2.8.3 Caco-2 cell culture

The permeation study was performed using as a model of the intestinal lining an *in vitro* method using Caco-2 cells differentiated simulating the intestinal barrier as previously described (Falé et al., 2013), using polycarbonate filters, 12 mm diameter, 0.4 µm pore diameter (Transwell^®^, Corning Inc. Lowell, MA, United States). Briefly, the Caco-2 cells at a density of 2 × 10^4^ cells/cm^2^ were seeded in 12-well transwell plate inserts with 10.5 mm diameter, 0.4 mm pore size (BD Falcon™). The differentiation of Caco-2 cells were verified by measuring the integrity of the monolayers was evaluated by measuring the transepithelial electrical resistance (TEER) with a Millicell ERS-2 V-Ohm Meter, from Millipore (Darmstadt, Germany), after 21 days of differentiation. The Caco-2 cells were considered differentiated when the TEER was higher than 250 Ω cm^2^. When differentiated, the cells were washed with HBSS and 0.5 mL of samples (dissolved in HBSS) were applied into the apical side of the transwell system. In the basolateral side (plate well) 1.5 mL of HBSS were added. The samples applied in apical side were caffeine (0.8 mg/mL), as a positive control and collagen peptides (0.5 mg/mL). High-Performance Liquid Chromatography (HPLC) Fingerprints was used to analyse the collagen peptides presented in apical and basolateral solutions at 0 and 6 h of incubation, as well as to analyse the caffeine solution used as a positive control. The HPLC analysis was performed in an Elite LaChrom^®^ VWR Hitachi liquid chromatograph (Tokyo, Japan) equipped with a Column oven L-2300 and Diode array detector L-2455 (VWR, United States). The column used was an Inertsil ODS C18 column (Thermo fisher). The HPLC conditions and gradient elution system was defined according to conditions described by Wang et al. (2018). To determine the total amount of peptides in both the apical and basolateral solutions, the areas of the peaks present in each chromatogram were added. The apparent permeability coefficient (Papp) was calculated from the permeation rate and compound concentration at 0 and 6 h of incubation, using the equation: Papp = [dQ/dt (A × Ci)] × (Cf × t).

Where V is the apical side volume in mL, A is the membrane area of the insert in cm^2^ (0.9 cm^2^), Ci is the initial concentration on the apical side (mg/mL), Cf is the concentration on the basolateral side (μM), and t is the time (s).

## 3 Results

### 3.1 Collagen extraction

Acid- and pepsin-soluble collagens (ASC and PSC) were successfully extracted and characterized from the scales of *S. pilchardus*. The yield of ASC was 0.18% (w/w) based on the dry weight of scales, while the yield for PSC was significantly higher, reaching 0.55% (w/w), approximately three times that of ASC. Similar studies have reported comparable yields from other fish species. For instance, Jaziri et al. (2022) reported ASC and PSC yields of 0.18% and 0.60%, respectively, from lizardfish (*Saurida tumbil*) scales. In a subsequent study, Jaziri et al. (2023) reported yields of 1.17% ± 0.19 for ASC and 1.00% ± 0.19 for PSC from parrotfish (*Scarus sordidus* Forsskål, 1775) scale waste. Kittiphattanabawon et al. (2019) reported yields of 0.77% for ASC and 0.71% for PSC from the scales of Nile tilapia (*O. niloticus*).

Moniruzzaman et al. (2019) investigated the extraction of collagen from carp (*Cyprinus carpio*) scales in different regions, reporting ASC and PSC yields of 0.97% and 1.37%, respectively for carp caught in Japan, and higher yields of 1.21% for ASC and 1.73% for PSC for carp from Bangladesh. They also found that lizardfish (*Saurida wanieso*) scales yielded of 0.44% for ASC and 0.72% for PSC in Japan, compared to 0.91% for ASC and 1.15% for PSC in Vietnam (Moniruzzaman et al., 2019).

The observed variation in collagen yields across different fish species may be influenced by factors such as extraction methods, tissue composition and structure, as well as differences in the size and age of the fish (Jaziri et al., 2023).

### 3.2 Characterization of collagen

#### 3.2.1 UV-Vis and FTIR spectra

**FIGURE 1:**
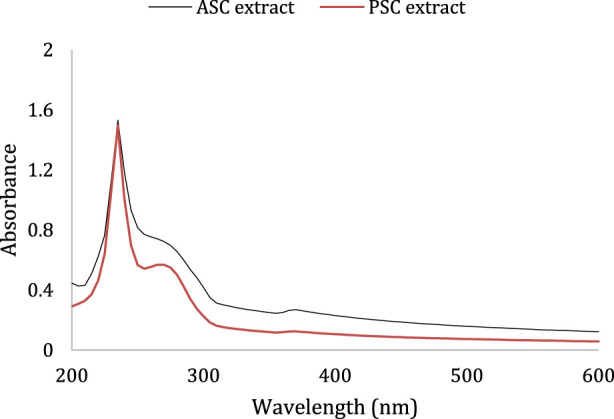
UV-Vis absorption spectra of ASC (acid soluble collagen) and PSC (pepsin soluble collagen) measured at a final concentration of 1 mg/mL of each sample from 200 to 400 nm.

The UV–Vis absorption spectrum serves as a crucial parameter for assessing collagen isolation efficiency, as the maximum absorption peak of collagen typically occurs at 230 nm, attributed to the C=O, -COOH, CONH_2_ groups within the collagen polypeptide chains (Jaziri et al., 2022). The results are shown in Figure 1 that illustrates the UV-Vis absorption spectra of the collagen extracted with acid (ASC) and with pepsin (PSC). The two collagen extracts exhibit maximum absorption peaks at 233 and 236 nm for ASC and PSC, respectively. This finding is consistent with Yu et al. (2018), who identified the peak absorption of collagen at 230 nm. Additionally, in the PSC, an absorption peak in the range of 250–300 nm is observed, indicative of impurities within the collagen peptide sample. Jaziri et al. (2022) elucidated this absorption peak in their study, attributing it to the presence of aromatic amino acids such as tryptophan (Trp) and tyrosine (Tyr), which are less abundant in collagen.

**FIGURE 2:**
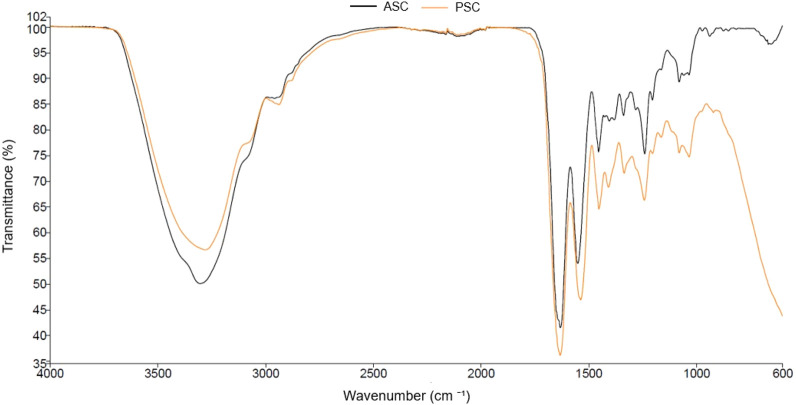
FTIR spectra of ASC (acid soluble collagen) and PSC (pepsin soluble collagen) freeze-dried extracts.

FTIR spectroscopy provides an easy way for researchers to explore functional groups and evaluate three-helix structure of collagen and its derivates (Ideia et al., 2020). The results of ASC and PSC extracts are shown in Figure 2, where the peaks indicate the presence of the characteristic groups present in collagen. The bands observed in the analyzed samples belong to Amide A, Amide I, Amide II, and Amide III.

Hernández-Ruiz et al. (2023) described the characteristic vibrations of amide groups as follows: Amide A vibrations (N–H stretching) occur between 3,270 and 3,310 cm^−1^, varying depending on the presence of hydrogen bonds; Amide B involves the asymmetrical stretch of CH_2_ within the region approximately between 2,850 and 3,100 cm^−1^; Amide I involve hydrogen bonding between N–H stretch and C=O within the range from 1,600 to 1,700 cm^−1^, while Amide II encompasses N–H bend and C–N stretching vibration spanning between 1,510 and 1,580 cm^−1^. Lastly, Amide III, characterized by C–N stretching and N–H deformation, falls within the range of 1,200 to 1,400 cm^−1^ (Hernández-Ruiz et al., 2023).

In the ASC sample, Amide A was observed at 3,304 cm^−1^, Amide I at 1,632 cm^−1^, Amide II at 1,551 cm^−1^, and Amide III at 1,239 cm^−1^. In other hand, PSC sample, Amide A was detected at 3,281 cm^−1^, Amide B at 2,939 cm^−1^, Amide I at 1,633 cm^−1^, Amide II at 1,537 cm^−1^, and Amide III at 1,241 cm^−1^. These findings validate that enzymatic treatment did not alter the inherent structure of collagen.

Similar findings in collagen extracted from marine samples have been reported by other researchers, including Abbas et al. (2022) and León-López et al. (2019b).

#### 3.2.2 Powder X-ray diffraction

**FIGURE 3:**
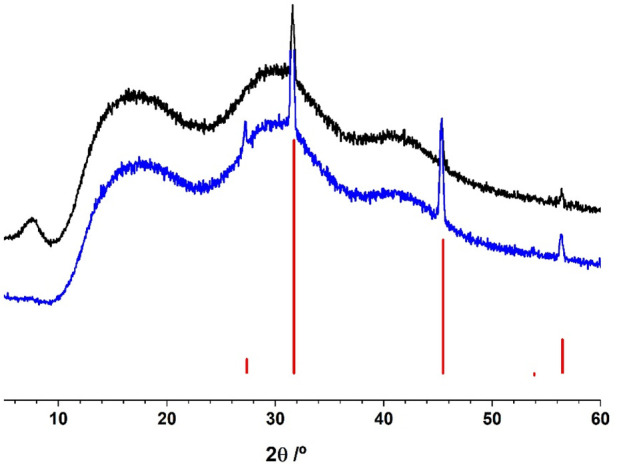
PXRD patterns for experimental ASC (acid soluble collagen) (black) and PSC (pepsin soluble collagen) (blue) samples and the NaCl pattern (COD 4300180) deposited in crystallography open database.

The X-ray diffraction diffractograms obtained from ASC and PSC extracts (Figure 3) were obtained under similar conditions. The main difference between both sampled concerns the presence in ASC sample of the broad peak that spans between 4.5° and 9.5° diffraction angles (2θ). According to the literature, this peak is associated with the triple-helical structure of collagen (Zhang et al., 2011; Giraud-Guille et al., 2000; Sun et al., 2017; Chen et al., 2016). The presence of this peak in ASC extract, indicates that the strength of the cross-linked pure collagen material and the density of collagen fiber molecular chains in the ASC extract is higher than in the PSC extract. This reinforces that ASC is a more ordered structure than PSC, justifying the hypothesis of cross-linked molecules (Tziveleka et al., 2022).

The second broad peak 11°–22° diffraction angles (2θ) is common to both ASC and PSC samples, and ii is due to the diffuse scattering triggered by many structural layers inside collagen fibers. The third peak found within the 26°–34° diffraction angles (2θ), similar in both samples, is related to the height of collagen triple-helical structural unit (Giraud-Guille et al., 2000).

Also in both samples, NaCl is detected (sharp peaks identified in Figure 3), and its identification was made recurring to the Crystallography Open Database. The PSC extract contains a higher amount of NaCl than ASC extract.

#### 3.2.3 Scanning electron microscopy (SEM)

**FIGURE 4:**
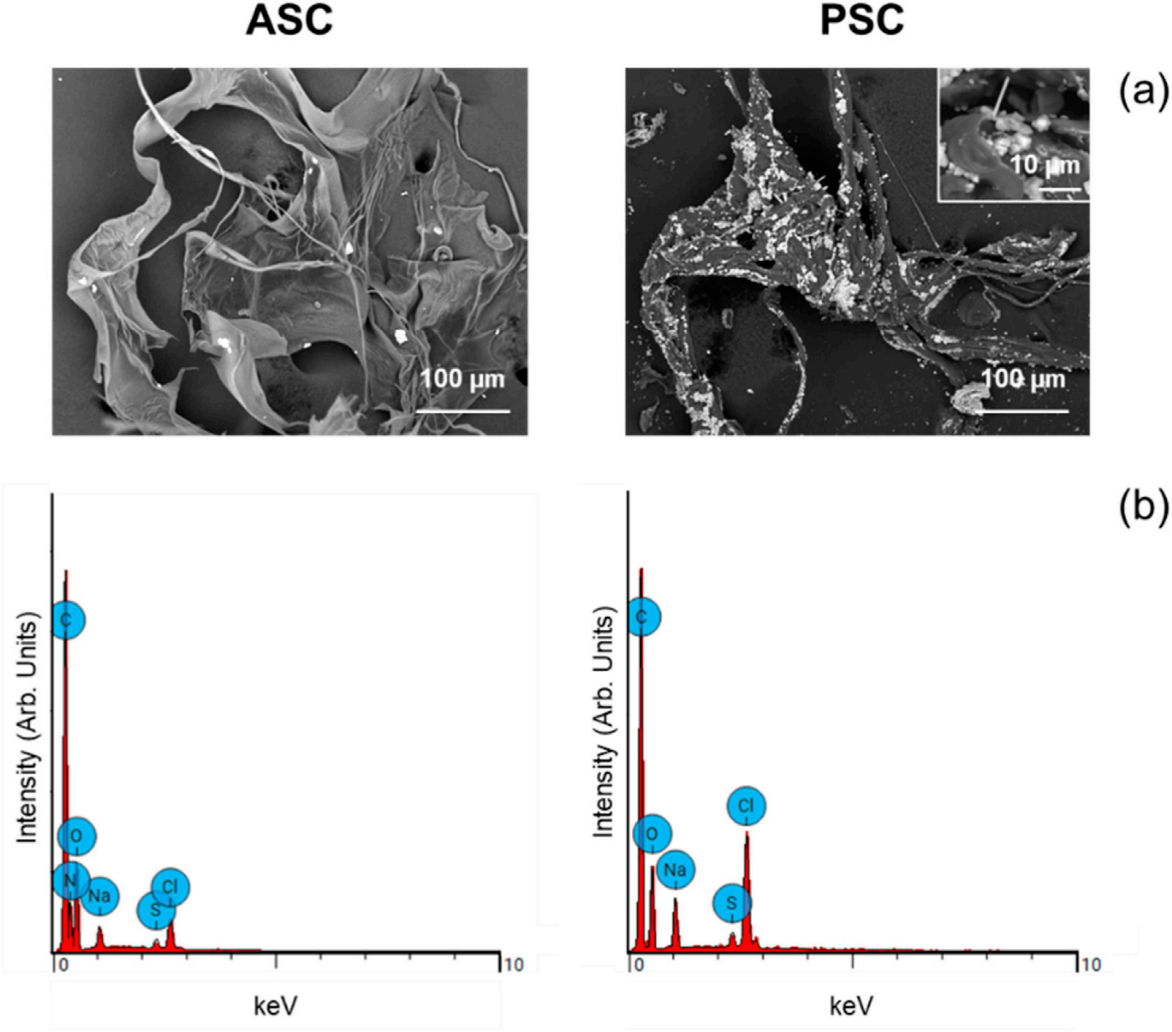
Physicochemical characterization of ASC (acid soluble collagen) and PSC (pepsin soluble collagen) extracts derived from *Sardina pilchardus* scales; **(A)** scanning electron microscopy (SEM) images, with the insert showing detailed PSC morphology, and **(B)** energy-dispersive X-ray spectroscopy (EDS) spectra.

A detailed physicochemical analysis by scanning electron microscopy (SEM), shows that both samples are uniform with larger and thinner stripes ranging from 5–100 µm (Figure 4A). Both ASC and PSC extracts have well-dispersed lighter structures in their polymeric matrix, with PSC harbouring a larger amount compared with ASC (Figure 4A). These entities, analysed by energy-dispersive X-ray spectroscopy (EDS) (Figure 4B), revealed the presence of Na and Cl. This data agrees with PXRD data where NaCl crystalline structures were depicted (Figure 3). Increased signal intensities for both Na and Cl, when compared with that of, agree with the higher number of crystals depicted in PSC sample compared with ASC (Figure 4). The presence of C, and O, is related to both the polymeric matrix and underneath carbon tape used in SEM analysis, whereas S links solely to the polymeric ASC and PSC matrixes (Figure 4B).

### 3.3 Enzymatic hydrolysis of collagen

#### 3.3.1 Analysis of ASC and collagen peptides by SDS-PAGE

**FIGURE 5:**
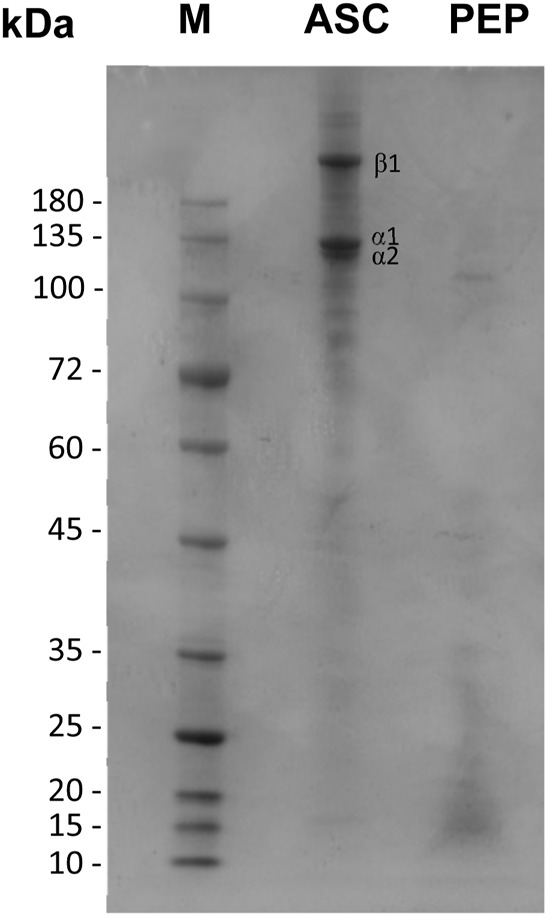
SDS-PAGE of collagen (ASC) and collagen peptides (PEP). Protein markers are depicted as M.

The protein pattern of ASC extract and collagen peptides (PEP) were analysed by SDS-PAGE electrophoresis (Figure 5), to confirm the type of collagen extracted and its purity, as well as to confirm the hydrolysis of collagen into small peptides. The protein pattern of ASC demonstrated its purity and the presence of three intense bands with, approximately, 200, 130, and 127 kDa, which can be attributed to β, α1, and α2 chains of type 1 collagen, respectively (Rabotyagova et al., 2008). The triple helix of type I collage is formed by two α1 chains, and one α2 chain, so the typical protein patterns of collagen Type I present the alpha 1 and alpha 2 bands in a 2:1 ratio among 120–150 kDa (Rabotyagova et al., 2008; Carvalho et al., 2018). The protein pattern of collagen type I also presents the β band that is a dimer with 200–250 kDA, corresponding to α1 and α2 chains together or two α -chains, and present the γ band, a trimer that represent tree alpha chains together (Carvalho et al., 2018; Gelse et al., 2003). In accordance with the typical protein pattern of collagen type I, the extracted collagen in this study presents the α1 band with higher intensity compared to α2 band (Figure 5). These results are also in accordance with previous studies with ASC extracted from scales of different fish species (Tziveleka et al., 2022; Rabotyagova et al., 2008).

The protein pattern of collagen peptides demonstrated the efficiency of the collagen hydrolysis method with papain, with no bands corresponding to the β and α chains being observed. Most of the protein bands of collagen peptides are found between 25 and 12 kDa, with a weak band of approximately 116 kDa also being observed.

### 3.4 Biological activities of collagen and collagen peptides

#### 3.4.1 Antioxidant capacity

The antioxidant activity of ASC extract and collagen peptides was assessed though the DPPH method. This method is the most frequently employed due to its quick and simple analysis (Hernández-Ruiz et al., 2023; León-López et al., 2019b). Nonetheless, reports on the antioxidant capacity of bioactive peptides are scarce (Hernández-Ruiz et al., 2023). Once the collagen was hydrolysed into small peptides, its antioxidant capacity increased by 3.6 times compared to the values obtained when analysed in its native form (Table 1).

**TABLE 1:** Antioxidant activity using DPPH method of collagen samples at 5 mg/mL.

Samples	% antioxidant activity
ASC extract	5.20 ± 0.38
Collagen peptides	18.91 ± 0.50
Positive control	95.35 ± 0.82

Positive control: quercetin at 0.1 mg/mL.

Previous studies demonstrated an association between the molecular weight of collagen and its antioxidant capacity (Hernández-Ruiz et al., 2023; León-López et al., 2019b). The IC_50_ of ASC was not defined due to its low antioxidant capacity, but the IC_50_ of collagen peptides was defined as 19.79 ± 0.85 mg/mL. Hernández-Ruiz et al. (2023) reported a IC_50_ values for DPPH of 48.83 ± 2.35 mg/mL for hydrolyzed collagen extracted from fish scales from tilapia (*Oreochromis aureus*). However, for a fraction of peptides with molecular mass between 5 and 10 kDa, they reported an IC_50_ value of 20.22 ± 0.75, a value similar to our result for collagen peptides. In another study, with a fraction of collagen peptides (10–30 kDa), González-Serrano et al. (2022) reported a percentage of antioxidant capacity of 70.93 ± 0.97, at 5 mg/mL, concluding that the antioxidant capacity of collagen peptides is influenced by the molecular weight and also by the presence of hydrophobic amino acids as glicine, alanine, or glutamic acid which contribute to greater antioxidant activity.

#### 3.4.2 Antimicrobial activity

The well diffusion method, a widely used screening technique for evaluating the antimicrobial potential of natural products (Balouiri et al., 2016), was used to assess the antimicrobial activity of the collagen and collagen peptides. The results indicated that none of the samples exhibited antimicrobial activity at a concentration of 10 mg/mL.

In a related study, Hernández-Ruiz et al. (2023) investigated the inhibitory effects of various collagen preparations of tilapia scales—pre-hydrolyzed collagen, hydrolyzed collagen, collagen peptides F1 (5–10 kDa), and collagen peptides F2 (<5 kDa)—on the growth of *E. coli* and *S. aureus* of clinical origin. The results demonstrated that for the *E. coli* strain, collagen peptides F1 induced the greatest inhibition of microbial growth. Specifically, the percent inhibition increased significantly to 50% for F1 compared to hydrolyzed collagen, indicating that fractionated collagen peptides with molecular weights of 5–10 kDa exhibit a superior antimicrobial effect. However, the growth of *S. aureus* was not inhibited by any of the collagen preparations (Hernández-Ruiz et al., 2023). Additionally, in the study by Lima et al. (2015), the peptide profile revealed several peptides with molecular weights lower than 2 kDa, which exhibited antibacterial activity against *E. coli*, *Bacillus subtilis*, and *S. aureus* (Lima et al., 2015). Similarly, the study of Song et al. (2012) tested the microbiological activity of peptides generated with different proteases and found that using pepsin to obtain peptides was the most effective. For *E. coli* CGMCC 1.1100, an inhibition zone of 11 mm was observed, while *S. aureus* CMCC 26003 showed an inhibition zone of less than 6 mm (Song et al., 2012).

It is important to nete that different strains of bacteria were tested in our study, which can influence the results. Additionally, our study found the protein bands of collagen peptides ranging between 12 and 25 kDa, and the size of the peptide can significantly impact their antimicrobial activity.

### 3.5 Permeation study with differentiated Caco-2 cells

To evaluate the ability of collagen peptides to permeate the intestinal barrier, Caco-2 cells grown and differentiated in the Transwell^®^ system for 21 days. After differentiation, Caco-2 cells present morphological and functional characteristics similar to small intestinal enterocytes (Ferruzza et al., 2012). This Caco-2 cell permeability assay is approved by the European Medicines Agency (EMA) and Food and Drug Administration (FDA) to evaluate the intestinal permeability of compounds (European Medicines Agency EMA, 2020; FDA, 2021).

**FIGURE 6:**
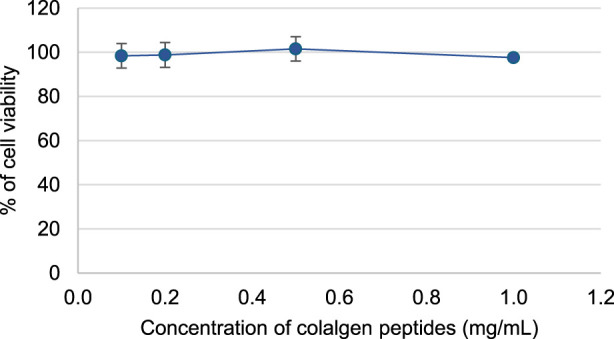
The effect of collagen peptides (in concentrations of 0.1, 0.2, 0.5 and 1 mg/mL) on Caco-2 cells viability.

Before the evaluation of collagen peptides capacity to permeate the intestinal barrier, the cytotoxicity effects of the peptide towards Caco-2 cells line was evaluated. At the different concentrations studied, collagen peptides do not present cytotoxicity effects (Figure 6). Similar results were previously reported with collagen peptides from tilapia scales (Lee et al., 2014).

**FIGURE 7:**
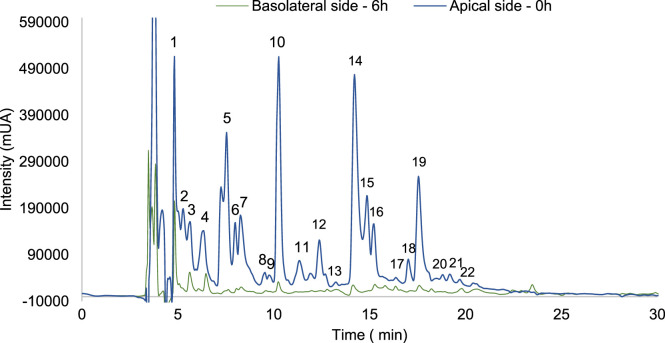
The high-performance liquid chromatography (HPLC) fingerprints of collagen peptides presents in apical solution at 0 h of incubation (–) and in basolateral solution after 6 h of incubation (–).

The collagen peptides (0.5 mg/mL) and caffein (0.8 mg/mL, positive control) were applied in the apical side of the Transwell^®^ system and incubated for 6 h, the normal range for small intestinal transit time (Lee et al., 2014). The fingerprint profiles of collagen peptides (Figure 7) and caffeine were analysed by Reverse Phase-High Performance Liquid Chromatography (RP-HPLC).

Following the international guidelines approved for permeability assays with Caco-2, the apparent permeability coefficient (Papp) values should be calculated to determine the permeability experimental values. The Papp value indicates the rate at which a substance is transported across the Caco-2 monolayer. A substance is classified as a low-permeability drug if its Papp is below 1.0 × 10^−^⁶ cm/s, as a moderate-permeability drug if its Papp is between 1.0 × 10^−^⁶ cm/s and 10 × 10^−^⁶ cm/s, and as a high-permeability drug if its Papp is above 10 × 10^−^⁶ cm/s. The Papp values obtained for collagen peptides and caffeine are resumed in Table 2.

**TABLE 2:** Apparent permeability coefficient (Papp: cm/s), apical to basolateral side, of collagen peptides (0.5 mg/mL) and caffeine (0.8 mg/mL) in Caco-2 permeability assay.

Samples	Papp (cm/s)
Collagen peptides	5.25 × 10^−6^
Caffeine	26.54 × 10^−6^

The Papp of caffeine demonstrates that it is a high-permeability drug, as previously described in the literature. On the other hand, collagen peptides have been shown to have moderate permeability. Similar results were previously obtained in a study on the intestinal permeability of bioactive peptides from collagen hydrolysates, with Papp values between 1 and 10 × 10^−^⁶ cm/s (Larder et al., 2021). It’s known that the peptides intestinal transport can occur through: paracellular transport, transcytosis, and carrier mediated transport (Renukuntla et al., 2013). The capacity of polypeptides to permeate through paracellular transport are inversely proportional to its molecular size, and also depends on their physicochemical properties, secondary structure and ionic charge (Renukuntla et al., 2013; Dodoo et al., 2000). Previous studies reported that some oligopeptides as well as di- and tripeptides can permeate the intestinal barrier by paracellular transport, being consider an important absorption pathway for peptides in intact forms (Quirós et al., 2008; Satake et al., 2002; Shimizu et al., 1997). Small contribution of transcytosis pathway has been described for hydrophobic and basic polypeptides (Satake et al., 2002; Sai et al., 1998). Most of the di and tri-peptides intestinal absorption has been described trough carrier mediated transport (Miguel et al., 2008). Shimizu et al. (2010) reported that size-dependent paracellular transport plays a major role in the transepithelial transport of fish collagen peptides across Caco-2 monolayers. Additionally, a previous study with collagen peptides obtained from tilapia scales reported a permeation rate of 32% after 2 h for peptides with 1 kDa, demonstrating that intestinal permeation of collagen peptides increased as molecular weight decreased (Je et al., 2019). These previous results may explain the moderate level of permeability observed with the collagen peptides under study, since as observed in the SDS-PAGE gel (Section 3.1), the peptides have masses between 25 and 10 kDa.

Previous *in vivo* studies have reported that hydrolyzed collagen oral supplementation has significantly improve skin conditions as: hydration, elasticity, firmness, reduce wrinkles, among others (Jhawar et al., 2020; Bolke et al., 2019; Addor et al., 2018; Kim et al., 2018), while also promoting orthopedic benefits such as increased bone strength, density, mass, improved joint mobility, and reduced pain (Wei et al., 2009; Bruyère et al., 2012; McAlindon et al., 2011).

Given the potential of collagen peptides as an oral supplement, it will be important to develop future studies to verify whether optimizing the hydrolysis process of collagen extracted from sardine scales leads to an increase in intestinal permeation of the collagen peptides.

## 4 Conclusion

In conclusion, the present study extracted and characterized acid-soluble (ASC) and pepsin-soluble (PSC) collagens from sardine scales (*S. pilchardus*), demonstrating their potential as sustainable sources of bioactive collagen. The yield of PSC (0.55%, w/w) was significantly higher than that of ASC (0.18%, w/w), which aligns with previous findings from similar studies on other fish species. The UV-Vis and FTIR spectra confirmed the presence of characteristic collagen functional groups, while the X-ray diffraction analysis indicated that ASC retains a more ordered, cross-linked triple-helical structure than PSC. This structural integrity of ASC suggests it may have higher mechanical strength and stability, whereas PSC exhibited a higher NaCl content, potentially due to the enzymatic extraction process.

Further physicochemical characterization using SEM and EDS revealed uniform polymeric matrices with dispersed NaCl crystals, particularly in PSC samples. SDS-PAGE analysis identified the presence of Type I collagen in ASC extracts, while collagen peptides showed smaller molecular weights, confirming the effective hydrolysis of collagen. The antioxidant activity of collagen peptides was markedly higher compared to native collagen, demonstrating a 3.6-fold increase in activity, likely due to their smaller size and the presence of hydrophobic amino acids.

Despite their moderate antioxidant capacity, neither the ASC nor the collagen peptides exhibited significant antimicrobial activity against the bacterial strains tested. However, the permeability assays using Caco-2 cells indicated that collagen peptides possess moderate intestinal permeability, suggesting potential for oral absorption and bioavailability. This aligns with the existing literature that shows the absorption of smaller peptides is favored due to their size and structural properties.

These findings highlight the potential of sardine scale collagen peptides in dermocosmetic and nutritional applications, particularly as oral supplements that could enhance skin health and provide orthopedic benefits. Future research should focus on optimizing the hydrolysis process to further increase the intestinal permeability and bioavailability of these collagen peptides, thereby expanding their utility in sustainable, health-promoting products.

## Data Availability

The raw data supporting the conclusions of this article will be made available by the authors, without undue reservation.
